# Quasi-Double-Blind Screening of Semiochemicals for Reducing Navel Orangeworm Oviposition on Almonds

**DOI:** 10.1371/journal.pone.0080182

**Published:** 2013-11-14

**Authors:** Kevin Cloonan, Robert H. Bedoukian, Walter Leal

**Affiliations:** 1 Honorary Maeda-Duffey Laboratory, University of California Davis, Davis, California, United States of America,; 2 Bedoukian Research Inc., Danbury, Connecticut, United States of America; Federal University of Viçosa, Brazil

## Abstract

A three-step, quasi-double-bind approach was used as a proof-of-concept study to screen twenty compounds for their ability to reduce oviposition of gravid female navel orangeworm(NOW), *Ameylois transitella* (Lepidoptera: Pyralidae). First, the panel of compounds, whose identity was unknown to the experimenters, was tested by electroantennogram (EAG) using antennae of two-day old gravid females as the sensing element. Of the twenty compounds tested three showed significant EAG responses. These three EAG-active compounds and a negative control were then analyzed for their ability to reduce oviposition via small-cage, two-choice laboratory assays. Two of the three compounds significantly reduced oviposition under laboratory conditions. Lastly, these two compounds were deployed in a field setting in an organic almond orchard in Arbuckle, CA using black egg traps to monitor NOW oviposition. One of these two compounds significantly reduced oviposition on black egg traps under these field conditions. Compound 9 (later identified as isophorone) showed a significant reduction in oviposition in field assays and thus has a potential as a tool to control the navel orangeworm as a pest of almonds.

## Introduction

Allelochemicals eliciting oviposition repellence and deterrence have been extensively studied for agriculturally important Lepidopteran pest species [1]–[13]. Several of these studies focused primarily on other Pyralid moth species [14]–[17].

The Navel Orangeworm (NOW*), Ameylois transitella*, (Walker) is the most serious insect pest [18]–[20] of the $3.6 billion dollar almond industry in California [21]. First instar larvae tunnel through the almond hull and into the nutmeat leaving behind larval frass and webbing as they develop into subsequent larval instars [22]–[25]. In addition to this direct feeding damage NOW infestation may also lead to infection of *Aspergillus* spp., which in turn produces aflatoxins [26]–[28].

Current management for NOW focuses primarily on winter sanitation, early harvest, and in-season insecticide applications [29], [30]. Other integrated pest management (IPM) practices include the use of biological control agents and mating disruption [27], [31]–[34]. Currently the combined effects of NOW infestation may greatly exceed 1% damage to the almond industry [27]. In an industry valued around $3.6 billion dollars, a 1% reduction results in millions of dollars of loss. Since 2002 the industry standard for NOW damage was less than 2% [19]. New approaches for control may be considered for a more robust IPM program of this most serious insect pest of almonds. Oviposition repellents and deterrents may provide, in conjunction with current IPM strategies, further control of the NOW in almonds.

In this proof of concept study, we screened a panel of 20 compounds, including known insect repellents, to determine if any of them would cause reduction of NOW oviposition under field conditions. This was a blind approach as we did not know the identity of the test compounds until the end of the experiments. Given that evaluation of 20 compounds under field conditions is tedious, time-consuming, and costly, we devised a three-step approach. We reasoned that active compounds would be detected by antennae of NOW gravid female moths [35]. Thus, non-electroangennogram (EAG) active compounds could be ruled out from an intermediate step: indoor two-choice behavioral bioassay. The field evaluation would then be performed with only a handful of promising compounds. As described here, we were able to reduce the panel to 3 compounds by EAG, then to 2 compounds by indoor behavioral bioassay, and finally tested 2 compounds in the field, one of which has practical potential applications in IPM strategies to control NOW populations.

## Materials and Methods

### Insects

The *A. transitella* moths used in this study were from a 2-year-old laboratory colony maintained at UC Davis. The UC Davis colony was initiated with moths kindly provided by Dr. Charles Burks, USDA-ARS (United States Department of Agriculture - Agricultural Research Service, Parlier, CA, USA) from his colony, which in turn was founded in 2005 [36]. At UC Davis larvae were reared in 1.5-L glass jars on a wheat bran, brewer's yeast, honey, and Vanderzant vitamins (Sigma-Aldrich, St. Louis, MO, USA) diet [37]. Jars were filled with 300 ml of diet to which ca. 300 eggs were added. Cultures were maintained in growth chambers (Percival Scientific, Perry, IA, USA) at 27°C, 70% RH, and a 16∶8 h (light:dark) photo regime. For colony maintenance newly emerged male and female moths were separated (ca. 50 males and ca. 50 females) into 12×12×5 cm plastic containers (Rubbermaid, Fairlawn, OH, USA) and lined at the bottom with one layer of moist paper towels and lined at the top with one layer of dry paper towels (Thirsty Ultra Absorbent, 27.9×27.9 cm; Safeway, Phoenix, AZ, USA) and left in rearing conditions for 72 h. After 72 h the top sheet containing red fertilized eggs was washed in a 10% formaldehyde solution for 15 min and rinsed with double-distilled water and allowed to air dry overnight. These eggs were then used for mass colony rearing.

### Electroantennogram (EAG) Recordings

For EAG assays last instar male and female larvae were separated into 1.5-L glass jars filled with 50 ml of artificial diet and allowed to pupate. Eclosed males and females of the same age were separated into 12×12×5 cm plastic containers (Rubbermaid, Fairlawn, OH, USA) and allowed to copulate. Copulation usually occurred the night following eclosion [20]. Mated pairs who mated on the first night after eclosion were separated into individually capped culture tubes (17 mm×10 cm; Fisher Scientific) and allowed to uncouple. Males were discarded. Female NOW moths are gravid and able to oviposit fertile eggs 24 h after mating [38]. For this reason two-day old mated females were used for all EAG and laboratory assays. We chose to use gravid females in all assays since this is the physiological state of females we would target in a field setting. The antennae of these females were excised and positioned on a fork electrode using an electrolytic gel and connected to an EAG Probe with an internal gain of 10×(Syntech, Kirchzarten, Germany). The EAG signals were recorded and analyzed with EAG 2000 software (Syntech). Antennal preparations were held in a constant stream of humidified air and stimuli delivery procedures were as previously described [39]. Briefly, stimulus pulses were delivered at a rate of 4 ml/s for 500 ms from a 5 ml polypropylene syringe containing a 2 mm strip of filter paper (70 mm diameter, Whatmanone Qualitative, GE Healthcare, Piscataway, NJ) impregnated with 10 µl of a test or control compound and recorded for 10 s. Each antennal preparation was stimulated with a battery of twenty test compounds at the same concentration: 10 µg/μl, 1 µg/μl, 0.1 µg/μl) and hexane as a control. The order of stimulation was randomized and a gap of 1 min was allowed between stimulations. Those antennal preparations that showed no response to our hexane controls were discarded and not included in our analysis. For each of the above concentrations three antennal preparations from three different females from three different cohorts were used.

### Small-Cage, Two-Choice Laboratory Oviposition Assays

Two-choice oviposition assays were performed under laboratory conditions and carried out in 30 cm×30 cm×30 cm green mesh cages (Bioquip, CA, USA). Each cage contained two-day old mated females (*N* = 20), isolated as above, and partitioned with a cotton ball soaked in a 10% sucrose solution on the floor of the cage. To monitor oviposition two black egg traps (Pherocon IV NOW, Black, Trece Inc., Adair, OK) were hung in opposite corners of each cage ca. 30 cm apart [36], [40]. These egg traps attract gravid female moths utilizing almond meal and almond oil as an attractant. The traps are lined with vertical grooves acting as substrate for the gravid moths to oviposit on. All black egg traps contained 1 g of larval diet to focus female oviposition [40]. Though no-choice assays have shown that in the absence of a preferred oviposition substrate female moths will oviposit on black egg traps alone [36], we wanted to focus our study on the effects these compounds have on the olfactory system of gravid female moths. Each black egg trap contained a 2 mm strip of filter paper (70 mm diameter, Whatmanone Qualitative, GE Healthcare, Piscataway, NJ) impregnated with either a control or a test compound. For all replicates control strips were impregnated with 10 µl of hexane. Test compound strips were impregnated 1 mg of test compound. We chose to use 1 mg of test compounds after several preliminary laboratory oviposition assays. These assays revealed the minimum dose eliciting a reduction in oviposition to be 1 mg. For each test compound 4 cages, each with 20 mated females, from three different cohorts of adult moths were tested for a total of 12 cages and 240 gravid females analyzed per test compound. To prevent any positional effects egg traps in all 4 cages for each trial were rotated clockwise to occupy a different location in each cage. All trials were setup 4 h before the scotophase and allowed to run for the length of the scotophase. All black egg traps were collected 2 h into the photophase and the number of eggs on each trap was counted. After each trial females were discarded and black egg traps, green mesh, and metal cage supports were then cleaned with hot water and Alconox, soaked in a 70% ethanol solution and allowed to air dry.

### Field Oviposition Assay

The almond orchard for this experiment was located at the Nickels Soils Lab (Arbuckle, CA, USA, University of California Cooperative Extension). The ca. 6 acre organic orchard chosen contained Nonpareil and Fritz varieties at a 3∶1 ratio with ca. 30 trees/row. This orchard was adjacent to a 23 years old ca. 20 acre conventional orchard containing: 33.3% Nonpareil, 33.3% Butte, 16.7% Carmel and 16.7% Monterey varieties. This field site was chosen for proximity to Davis, CA as well as a history of NOW infestation and little chemical control (personal communication, F. Niederholzer, UC Extension). The experimental plot consisted of two perpendicular border rows, one West facing and the other South facing. Nonpareil trees comprise the majority of the almond acreage planted in California [19], [21]. For this reason only Nonpareil trees were used in the experiment. Every fifth or sixth Nonpareil tree was chosen for the experiment starting from the West facing row and continuing down the South facing row for a total of 18 trees. The trunk of each tree was vertically fixed with a 1.8 m PVC pipe (10 mm in diameter, Ace Hardware, Davis, CA, USA) and fastened with a 0.3 m long bungee cord. To this another 1.8 m PVC pipe was attached for a total height of ca. 3.6 m. These second 6 ft1.8 m PVC pipes had three holes (1 mm in diameter) drilled ca. 0.3 m apart. The wire end of the black egg traps were then secured into these holes to prevent them from falling out of the trees. Each of these 1.8 m PVC pipes was fixed with either two or three black egg traps depending on the experiment. All black egg traps were filled with 15 g of almond meal, (Pherocon® IV Bait,Trece Inc., Adair, OK, USA) commonly used to monitor ovipositing female NOW moths in the field [36], [40]–[42]. Nestled in the center of the almond meal of each egg trap was a rubber septa impregnated with 10 mg of either a control or test compound (Precision Seal® rubber septa red, 8 mm O.D. glass tubing; Sigma-Aldrich, St. Louis, MO, USA). Each day between 9:00 AM and 12:00 PM Pacific Standard Time (PST) NOW eggs on each black egg trap were counted and destroyed with a toothbrush. The positions of egg traps on the last segment of 1.8 m PVC pipe were then rotated. Black egg traps, almond meal, and rubber septa were changed every three days. Black egg traps were cleaned with hot water and Alconox, soaked in a 70% ethanol solution and allowed to air dry after use in the field.

To determine the biofix, or first of two days with 75% increase in detected oviposition, 18 black egg traps filled only with 15 g of almond meal and were hung on the 18 experimental trees in the organic block and checked every other day starting April 1st, 2012. Black egg traps and almond meal were replaced on a weekly basis. The biofix was recorded on May 9th, 2012 as the first of two dates with a 75% increase in eggs [30].

For the first trial all materials were placed in the field on May 12th, 2012 and pulled from the field on June 23rd, 2012 following two weeks of no egg detection. Each of the 18 trees was a single competitive assay between compounds 8, 9, and hexane, all vertically spaced 60 cm apart on the PVC pipe. Each of the 18 trees contained three black egg traps on the second 1.8 m PVC pipe. Each black egg trap contained a rubber septa impregnated with 10 mg of either compound 8 or compound 9; control had hexane only.

After this first trial each of the 18 trees was once again fixed with just black egg traps and 15 g of almond meal and treated as above to determine when oviposition could once again be detected. NOW eggs were detected again on September 10th, 2012.

For the second trial all materials were placed in the field on September 13th, 2012 and pulled from the field on October 18th, 2012 following two weeks of no egg detection. Each of the 18 trees was a two-choice assay between compound 9 and hexane, vertically spaced 120 cm apart on the PVC pipe. Each of the 18 trees contained two black egg traps on the second 1.8 m PVC pipe. Each black egg trap contained a rubber septa impregnated with 10 mg of either compound 9 or hexane as a control.

### Chemical Preparation

The panel of compounds, which was decoded at the end of the field results, was prepared at Bedoukian Research Inc (BRI, Danbury, CT, USA). It includes 20 compounds covered by US Patent Application No. 61/687,920 for application on vegetation. Compounds were originally discovered when testing compounds closely related to naturally occurring ketones and lactones, finally focusing in on cyclic ketones and lactones and includes:

**1**: Farnesyl cyclopentanone, **2**: (*E,E*)-farnesol, **3**: methyl dihydrojasmonate, **4**: methyl jasmonate, **5**: γ-decalactone, **6**: δ-tetralactone, **7**: ethyl palmitate, **8**: isophrol, **9**: isophorone, **10**: prenyl dihydrojasmonate, **11**: 2-pentadecanol, **12**: 3,5,5-trimethylcyclohexanol, **13**: methyl apritol, **14**: methyl dihydrojasmolate, **15**: dihydrojasmonic acid, **16**: methyl apritone ( = miranone), **17**: dihydrojasminlactone, **18**: dihydrojasmindiol, **19**: ethyl dihydrojasmonate, and **20**: 2-pentadecanone. All test compounds were diluted in hexane to make stock solutions of 100 µg/μl. Decadic solutions were then made in hexane for desired concentrations of 10 µg/μl, and 1 µg/μl and 0.1 µg/μl. The 2 mm strips of filter paper used in both the EAG and laboratory ovipositon assays were allowed to air dry under a fume hood for 2 min before use. For field ovipositon assays 100 µl of the 100 µg/μl stock solution were used for application of 10 mg in rubber septa. This volume was allowed to soak into the rubber septa for 1 h before nestled inside the black egg traps.

### Statistical Analysis

To analyze EAG recordings we did not normalize our data [43] because the variation between individual antennal preparations was low. Mean responses for all compounds were compared to the hexane control within all concentrations. All data were first tested for normality via the D'Agostino-Pearson omnibus test. Data were then analyzed with either ANOVA or the Kruskal-Wallis test using GraphPad Prism v5.0 software (GraphPad Software Inc., CA, USA). For both the laboratory and field oviposition assays mean eggs per trap were analyzed. Since each tree in the field assay contained all treatments each tree was considered an experimental unit. Those trees that had no black egg traps with eggs were excluded from each day analysis and therefore the final analysis.

## Results

This proof of concept study was approached in a quasi-double-blind test. Although we knew the controls, the identity of the test compounds was unknown to the investigators at UC Davis until the end of the field tests. The panel of 20 compounds was prepared at BRI and the experimenters were aware that it included insect repellents and non-insect repellents (placebos). To make the preparer blind, we changed their code into compound numbers. Here, we will initially describe the results with compound numbers (as it happened during the tests) and decode them at the end of the section.

**Figure 1:**
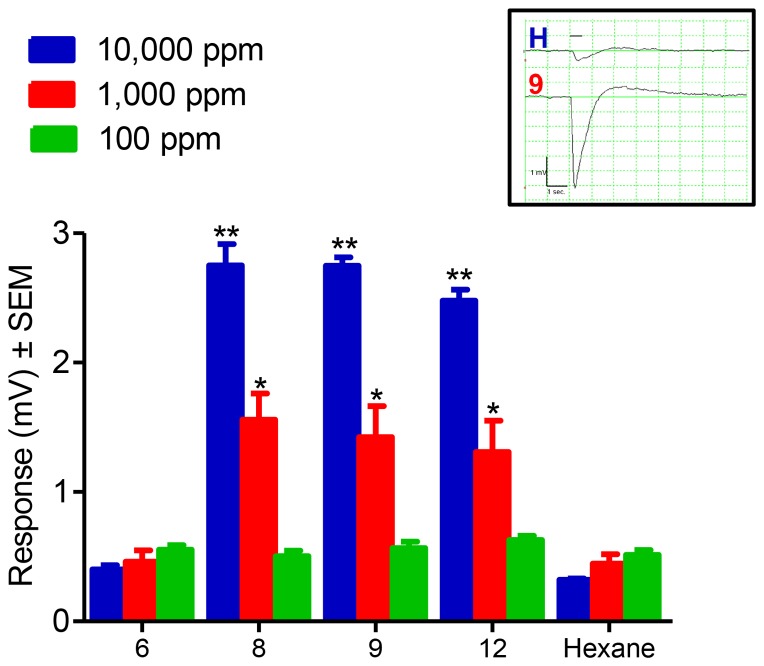
EAG responses from gravid female moths to test compounds. EAG responses recorded from two-day old mated female NOW moth antennae. Dose dependence responses (*N* = 9) for compounds **6**, **8**, **9**, and **12** at concentrations 10 µg/μl (≈10,000 ppm), 1 µg/μl (≈1,000 ppm), and 0.1 µg/μl (≈100 ppm). There were significantly higher responses for compounds **8**, **9**, and **12** as compared to the hexane controls at concentrations 10 µg/μl (*P* = 0.0001; Tukey's Multiple Comparison Test) and 1 µg/μl (*P* = 0.0001). EAG responses at concentration of 0.1 µg/μl were not significantly different (*P*>0.05) from hexane controls. Compound **6** did not show EAG activity (*P*>0.05). *Inset* Representative EAG traces of hexane control (H) and compound **9** (9) at 10 µg/μl. The solid line above the traces represents stimulus duration (500 ms). The time and voltage scales are shown at the bottom left of the graph.

Initially we screened the 20 compounds via EAG using gravid, two-day old female NOW moth antennae as the sensing element. Of the three concentrations that were tested (10 µg/μl, 1 µg/μl, and 0.1 µg/μl) the greatest responses were recorded at 10 µg/μl. Of the twenty compounds tested compounds **8**, **9**, and **12** showed significantly higher EAG responses compared to the hexane control (*P*<0.05; *P*<0.05; *P*<0.05; respectively) for 10 µg/μl and 1 µg/μl (Fig. 1).

**Figure 2:**
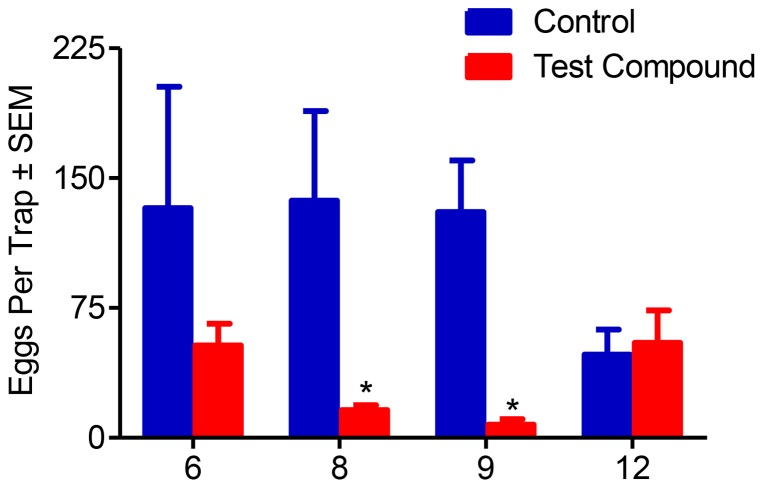
Small-cage two-choice laboratory oviposition assay. For each compound tested there were four cages each with twenty two-day old mated female NOW moths replicated three times from three different cohorts (*N* = 12). Gravid female moths laid significantly fewer eggs on those black egg traps treated with compound **8** (*P* = 0.03) as well as compound **9** (*P* = 0.003; analyzed via the Mann-Whitney Test) when compared to traps treated with hexane (controls). No significant differences were observed for oviposition on black egg traps spiked with compound **6** (*P* = 0.95) or compound **12** (*P* = 1.0) as compared to control traps.

Next we examined the effect of EAG-active compounds on the oviposition of female NOW moths in small-cage, two-choice laboratory assays. For these experiments we tested compounds **6**, **8**, **9**, and **12**. Compounds **8**, **9**, and **12** all showed significantly greater EAG responses compared to the hexane controls (Fig. 1). From those compounds that did not show a significant EAG response compared to the hexane control (Fig. 1) we chose compound **6** to serve as a negative control. There was a significant difference of mean eggs per trap between those black egg traps containing filter papers impregnated with hexane and those containing filter papers impregnated with compound **8** (*P*<0.005). Similar results were seen for compound **9** (*P*<0.02). There was no significant difference of mean eggs per trap between those black egg traps containing filter papers impregnated with hexane and those containing filter papers impregnated with compound **12** (*P* = 0.80). Similar results were observed for compound 6 (*P* = 0.74) (Fig. 2).

**Figure 3:**
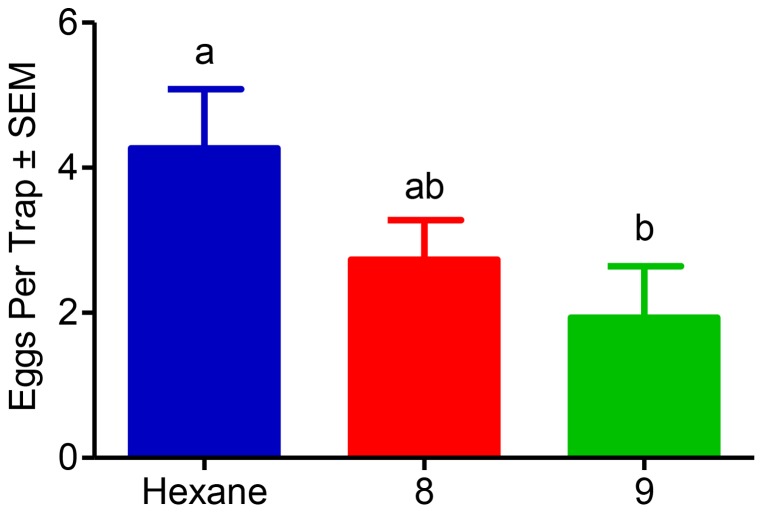
Multiple-choice field evaluations of compounds pre-screened by EAG and indoor bioassay. This first trial was conducted in Arbuckle, CA from May 13th, 2012 through June 9th, 2012. The number of eggs laid on black egg traps baited with compound **9** was significantly lower than those oviposited on control (hexane) traps (*P* = 0.003; Dunn's Multiple Comparisons Test). Oviposition in traps baited with compound **8** was not significantly different (*P*>0.05) from those on control traps. Lastly, there was no significant difference between the two treatments: compounds **8** and **9** (*P*>0.05; *N = 30*).

Lastly we tested the effect of those compounds that showed significantly reduced oviposition from the small-cage two-choice laboratory assay to female NOW moths in a field setting. The first trial ran for 27 days from May 13th, 2012 through June 9th, 2012. Each of the 18 trees in this first trial was a competitive assay between hexane, compound **8**, and compound **9**. Oviposition events were monitored 30 times on 15 of the 18 trees during these 27 days. Ovipositional events can be defined as newly laid eggs on an egg trap within a 24 h period. There was no pattern of ovipositional preference between the 18 trees during this 27 day period. There was no significant difference of mean eggs per trap between those black egg traps containing rubber septa impregnated with hexane and those containing rubber septa impregnated with compound **8** (*P* = 0.16). There was a significant difference of mean eggs per trap between those black egg traps containing rubber septa impregnated with hexane and those containing rubber septa impregnated with compound **9** (*P* = 0.0002) (Fig. 3).

**Figure 4:**
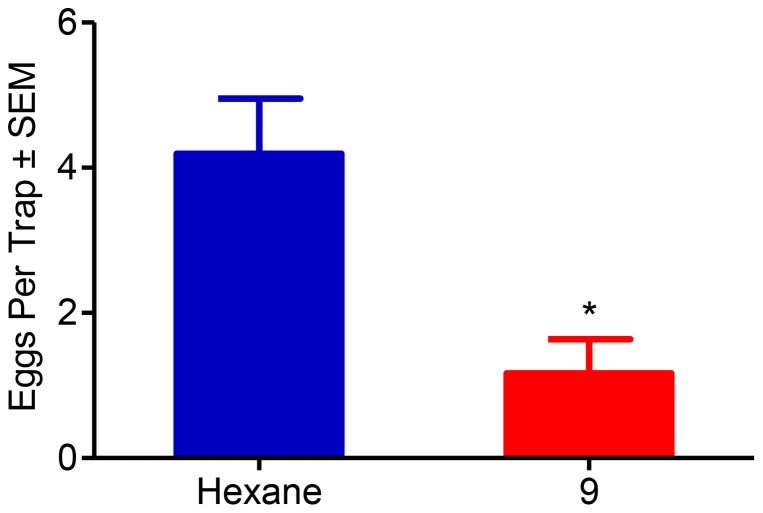
In-depth field evaluations of an active compound. This second field trial was performed in Arbuckle, CA from September 14th, 2012 through October 18th, 2012. In these direct comparison compound **9** was highly significant (*P* = 0.0001; Mann-Whitney Test, *N = 34*).

The second trial of this field experiment ran for 34 days from September 14th, 2012 through October 18th, 2012. Each of the 18 trees in this second trial was a two-choice assay between hexane and compound **9**. We chose to exclude compound **8** from this second trial to minimize or avoid intertrap competition. Oviposition events were monitored 36 times on 14 of the 18 trees during these 34 days. There was no pattern of ovipositional preference between the 18 trees during this 34 day period. There was a significant difference of mean eggs per trap (*P*<0.0001) between those black egg traps containing rubber septa impregnated with hexane and those containing rubber septa impregnated with compound **9** (Fig. 4).

Once we discussed the field test results, the preparer (R.H.B.) disclosed the code names he used for the 20 test compounds, and the experimenters (K.R.C. and W.S.L.) matched these with their own compound numbers to identify the complete panel of test compounds. Thus, compound **6** is δ-tetradecalactone (CAS# 2721-22-4), **8**, isophorol (CAS# 470-99-5), **9**, isophorone (CAS# 78-59-1), and **12** is 3,5,5-trimethylcyclohexanol (CAS# 116-02-09); the complete list is provided in Methods and Materials section. Therefore, our study suggests that isophorone has potential practical applications in IPM strategies as it reduced oviposition by the navel orageworm under field conditions.

## Discussion

Our ultimate goal was to identify semiochemicals that effect the oviposition of NOW in a field setting. Since deploying these twenty compounds in the field would prove cumbersome and inefficient, we developed a 3-step screening method to identify those behaviorally significant compounds. First, through EAG analysis, we identified three of the twenty compounds that gravid female moth antennae could detect using female moth antennae as the sensing element. Next, using small-cage, two-choice assays, we examined the effect of these three compounds on female NOW oviposition. Of these three compounds two significantly reduced oviposition under these laboratory conditions. As was our ultimate goal, we then deployed these two compounds in the field in Arbuckle, CA to examine their effects on female oviposition. After two field trials in 2012 we found that isophrone ( = compound **9**) significantly reduced oviposition under field conditions, thus showing tremendous potential to control NOW populations in almonds.

The mean eggs per trap for both of the field experiments were lower compared to previous studies using black egg traps to monitor NOW oviposition [27], [36], [44]. This is likely due to the lower accumulation of degree days in more Northerly almond orchards as compared to more Southerly orchards [30], [45], [46] leading to a condition of reduced NOW development and abundance in our Northern field site. Though our mean eggs per trap counts were low, previous work has shown that at low mean eggs per trap the presence or absence of eggs on black egg traps is more important for monitoring the behavior of female NOW oviposition than the actual number of eggs per trap [41].

Previous research has shown allelochemicals that reduce oviposition in other agriculturally important pyralid moth species [14]–[17]. There is an ever-increasing demand for these reduced-risk insecticides in agriculture [47]–[49]. Due to their low mammalian toxicity, non-lethal effects, and high selectivity to insects, repellent and deterrent allelochemicals may present a viable reduced-risk addition to current IPM practices for NOW as a pest of almonds [49], [50].

For future work we would like to examine the longevity of isophorone ( = compound 9) under field settings and higher NOW densities, potentially in more Southern almond orchards of California. The hope is that behaviorally significant compounds, identified through these described screening methods, will eventually be used in large-scale field experiments. Phytotoxitity experiments should be coupled with behavioral assays in the field as previously described [51]. As a generalist scavenger of stonefruit and nut crops [25], it may be the case that the insect is able to identify a suite of plant produced semiochemicals as host cues, and can discriminate different blends of these semiochemicals as host attractants. If this is the case, it may also be likely that the insect can perceive and respond to a blend of plant produced semiochemicals to a greater degree than single plant compounds. There then lies potential to combine compounds showing repellency in a mixture that may show an even greater reduction in oviposition than compound 9 alone. For later field assays in more southern orchards we would also like to examine the ability of compound 9 to reduce oviposition in no-choice assays against hexane controls in egg traps. In these future trials, one tree should be fixed with a PVC pipe as described above and only one egg trap, impregnated with hexane or compound 9, and compared with an adjacent tree fixed with the alternative treatment. We would also like to examine the intriguing question of what behavioral modality these compounds elicit in the navel orangeworm. Are these compounds acting as oviposition repellents or oviposition deterrents [52]? Though very interesting and important scientific questions, this study sought to develop a series of assays one could use to viably and efficiently screen many compounds to identify those that can reduce oviposition of the navel orangeworm.
